# A One Medicine Mission for an Effective Rabies Therapy

**DOI:** 10.3389/fvets.2022.867382

**Published:** 2022-03-16

**Authors:** Darryn L. Knobel, Alan C. Jackson, John Bingham, Hildegund C. J. Ertl, Andrew D. Gibson, Daniela Hughes, Kenneth Joubert, Reeta S. Mani, Bert J. Mohr, Susan M. Moore, Hugh Rivett-Carnac, Noël Tordo, James W. Yeates, Anthony B. Zambelli, Charles E. Rupprecht

**Affiliations:** ^1^Department of Biomedical Sciences, Ross University School of Veterinary Medicine, Basseterre, Saint Kitts and Nevis; ^2^Department of Veterinary Tropical Diseases, Faculty of Veterinary Science, University of Pretoria, Pretoria, South Africa; ^3^Canine Rabies Treatment Initiative, Salt Rock, South Africa; ^4^Department of Medicine, Northern Consultation Centre, Thompson General Hospital, Thompson, MB, Canada; ^5^Department of Medicine, Lake of the Woods District Hospital, Kenora, ON, Canada; ^6^Commonwealth Scientific and Industrial Research Organisation (CSIRO) Australian Animal Health Laboratory at the Australian Centre for Disease Preparedness, Geelong, VIC, Australia; ^7^Wistar Institute, Philadelphia, PA, United States; ^8^Division of Genetics and Genomics, Easter Bush Veterinary Centre, The Roslin Institute and the Royal (Dick) School of Veterinary Studies, The University of Edinburgh, Roslin, United Kingdom; ^9^Veterinary Anaesthesia, Analgesia and Critical Care Services, Lonehill, South Africa; ^10^Department of Neurovirology, WHO Collaborating Centre for Reference and Research in Rabies, National Institute of Mental Health and Neurosciences, Bangalore, India; ^11^Centre for Animal Research, Faculty of Health Sciences, University of Cape Town, Observatory, South Africa; ^12^Veterinary Medical Diagnostic Laboratory, University of Missouri, Columbia, MO, United States; ^13^Institut Pasteur de Guinée, Conakry, Guinea; ^14^Independent Researcher, Midhurst, United Kingdom; ^15^LYSSA LLC, Atlanta, GA, United States

**Keywords:** rabies, treatment, pathogenesis, prognosis, canine, neurodegeneration, immunotherapy, blood-brain barrier

## Abstract

Despite the disease's long history, little progress has been made toward a treatment for rabies. The prognosis for patient recovery remains dire. For any prospect of survival, patients require aggressive critical care, which physicians in rabies endemic areas may be reluctant or unable to provide given the cost, clinical expertise required, and uncertain outcome. Systematic clinical research into combination therapies is further hampered by sporadic occurrence of cases. In this Perspective, we examine the case for a One Medicine approach to accelerate development of an effective therapy for rabies through the veterinary care and investigational treatment of naturally infected dogs in appropriate circumstances. We review the pathogenesis of rabies virus in humans and dogs, including recent advances in our understanding of the molecular basis for the severe neurological dysfunction. We propose that four categories of disease process need to be managed in patients: viral propagation, neuronal degeneration, inflammation and systemic compromise. Compassionate critical care and investigational treatment of naturally infected dogs receiving supportive therapy that mimics the human clinical scenario could increase opportunities to study combination therapies that address these processes, and to identify biomarkers for prognosis and therapeutic response. We discuss the safety and ethics of this approach, and introduce the Canine Rabies Treatment Initiative, a non-profit organization with the mission to apply a One Medicine approach to the investigation of diagnostic, prognostic, and therapeutic options for rabies in naturally infected dogs, to accelerate transformation of rabies into a treatable disease for all patients.

## Introduction

Rabies is one of the oldest described infectious diseases of humans and dogs (1). An entry in the *Susruta Samhita*, a text from around 600–1,000 years BCE, states that if a person bitten by a rabid dog shows signs of hydrophobia—a feature characteristic of rabies in humans—the person is considered “doomed” (2). Despite considerable advances in knowledge of the etiology and pathogenesis of this neglected tropical disease (NTD) over the intervening millennia, the prognosis for human (and canine) patients with clinical manifestations of rabies remains largely unchanged today. This zoonosis has the highest case fatality of any infectious disease, with death typically occurring within 14 days of onset of clinical signs. Since the 1970s, survival has been well documented in only around 30 of the estimated 3 million human cases that have occurred in that time (3–5). Of these survivors, most suffered severe neurological sequelae (3, 4).

A report of the recovery, with only minor neurological deficits, of a previously unvaccinated rabies patient following induction of a therapeutic coma in Milwaukee, WI, USA in 2004 provided hope of an effective therapeutic protocol (6). Unfortunately, multiple efforts to replicate this Milwaukee protocol have been unsuccessful, with at least 53 documented failures (3). Although there has been an increase in the number of reports of survival from rabies in more recent years, particularly from India, this is likely due to improvements in critical care and more attempts at aggressive management rather than development of specific therapies. Survivors frequently have severe neurological sequelae, with a poor quality of life (3, 7, 8). With abandonment of the Milwaukee protocol, and with poor patient prognoses even with aggressive management, physicians are presented with few therapeutic options beyond palliative care (4, 9).

Worldwide, most human rabies deaths are caused by infection with rabies virus (RABV) transmitted from domestic dogs. Accurately determining the number of people who die from dog-transmitted rabies is hampered by lack of reliable surveillance data from many of those countries where the disease is most prevalent, but estimates place this number at ~59,000 each year (95% confidence intervals 29,000–159,000) (5). The burden of this NTD falls predominantly on populations in resource-poor communities in Africa and Asia. The number of dogs that die from rabies each year is unknown, but extrapolation of figures used to estimate the burden of human disease would place this number in the millions, if not tens of millions. This number does not include those dogs that are not rabid but are killed on suspicion of being infected or in response to rabies outbreaks.

The fatal outcome of rabies and lack of therapeutic options have focused efforts on prevention of human cases by reducing or eliminating transmission of RABV among dogs through mass vaccination, and through the timely provision of post-exposure prophylaxis (PEP) to exposed people. These efforts are culminating in the goal to eliminate human deaths from dog-transmitted rabies by 2030 (10). Alongside this, efforts to develop a treatment for this NTD should continue. Rabies is not a candidate for eradication. The disease will continue to pose a threat in circumstances in which there is failure to recognize exposure or to seek PEP; when there is lack of access to PEP or deviation from recommended PEP protocols; or in rare cases when PEP fails or is refused (11, 12). Transmission of RABV from wildlife hosts will continue to pose a risk, including for the re-emergence of dog rabies through spillover events and host switching (13). Lyssaviruses against which PEP is not effective continue to emerge and pose a threat to public and animal health (14, 15).

Thus, there remains a need to develop an effective therapy for rabies. This paper examines the case for a One Medicine approach to research and development of a combination therapy in naturally infected canine patients, to supplement research in experimental animal models and human cases. We conclude with a call to action for veterinarians to play a more active role in development of an effective therapy for this NTD.

## Rabies Virus Pathogenesis

The pathophysiology of rabies has been reviewed in detail (16–18). Many of the clinical features of rabies are similar in human and canine cases, although not all elements are well described in both species. The main mode of transmission of RABV in both hosts is through exposure of mucosal membranes or broken skin to the saliva of an infected dog, typically through a bite, scratch, or lick. Clinical signs develop after a variable and occasionally prolonged incubation period, and are often non-specific, particularly in the early stages of disease. As the disease progresses to the acute neurological phase, one of two clinical forms usually become apparent. Most human and canine patients develop an encephalitic or “furious” form, while the remainder develop a paralytic or “dumb” form (18, 19). The pathogenesis of the two forms is unclear, although the host immune response is thought to play an important role, with a more prominent inflammatory response evident in paralytic cases (20, 21). Both forms of the disease typically progress to coma and death within days, although the time to death may be influenced by critical care interventions.

A typical feature of rabies is that despite the consistent and catastrophic clinical outcomes, histopathological changes observed in the central nervous system (CNS) after death following acute disease are relatively mild. Preservation of the neuronal network is essential for this strictly neurotropic virus to complete its cycle within a host, from site of entry, transit, and propagation through the nervous system, and exit through the salivary glands. As such, RABV has evolved mechanisms to promote survival of infected neurons by inhibiting apoptotic death and by avoiding or inhibiting activation of inflammatory responses that would lead to death of infected cells (22–25). Rabies virus neutralizing antibodies (RVNAs) are often detectable only late in the course of disease, if at all (7, 18, 26). There is evidence that RABV infection of the CNS maintains the blood-brain barrier (BBB), thereby minimizing viral exposure to circulating RVNAs (27, 28). Permeability of the BBB may be affected by the animal model and RABV isolate used. Given the importance of the BBB for effective drug delivery (see Therapeutic Approaches), its permeability in naturally infected cases warrants investigation.

Recent findings have advanced our understanding of the pathological basis for the severe neurological dysfunction that occurs in rabies and may open new avenues for treatment. Although conventional histopathology reveals few changes, ultrastructural studies in a mouse model showed severe degeneration of neuronal processes, characterized by swelling and/or beading of axons in particular (29). These changes are considered sufficient to explain the severe clinical disease and fatal outcome in rabies (29, 30). Changes are associated with oxidative stress resulting from mitochondrial dysfunction (30–32). Sundaramoorthy et al. (33), using an *ex vivo* neuronal model, identified the enzyme SARM1 as the central mediator of this axonal degeneration in RABV-infected neurons. When activated, the TIR domain of SARM1 breaks down nicotinamide adenine dinucleotide (NAD^+^). The SARM1 enzyme, normally maintained in an inhibited state, is activated by axon injury or disease (34). This results in a rapid loss of NAD^+^ that is sufficient to drive axon degeneration (35), through an ordered process of loss of cellular ATP, mitochondrial dysfunction, calcium influx and loss of membrane permeability (36). In RABV infection, SARM1-mediated axonal degeneration impedes the spread of virus among interconnected neurons, but also results in the pathological loss of axons and dendrites that could explain the severe neurological dysfunction in late-stage rabies (33). Future studies should determine whether this pathway of axonal degeneration occurs in naturally infected hosts and investigate upstream initiators of SARM1 activation. One candidate for the latter is the mitochondrial dysfunction seen in RABV infection that results from interaction of the viral phosphoprotein with mitochondrial complex 1 in infected neurons (37). Notably, while mitochondrial dysfunction is a consequence of programmed axon death following SARM1 activation, it has also been identified as an important upstream initiator of the process (38).

## Therapeutic Approaches

Given the complexity of the pathological processes following RABV infection of the CNS and the severity of the outcome, it is likely that combination therapies will be needed for successful treatment, targeting different aspects of the disease process (3, 39, 40). We propose that the following four broad categories of disease process need to be managed to ensure the best patient outcome:

### Viral Propagation

Inhibition of viral propagation using specific antiviral drugs or immunotherapeutics will be essential. This could target different steps of cell infection including receptor recognition, endosomal pathway, virus replication, intra-neuronal transport or cell budding. Antiviral drugs for use against RABV have been reviewed (41–44). Despite promising *in vitro* and occasionally *in vivo* results, no drugs have shown clinical benefits in patients. Development of an effective antiviral agent for therapy of rabies remains an important goal for the future.

Immunotherapeutics, in the form of monoclonal antibodies (mAbs) targeting specific RABV proteins, offer some promise. Single (SII RMab) and cocktail (docaravimab and miromavimab) mAb products against the RABV glycoprotein are safe and effective for rabies PEP given shortly after exposure (45, 46). Access of antibodies to sites of viral replication in the clinical phase of the disease, when RABV is widely distributed in the CNS, is however problematic. Recently, de Melo et al. (47) showed that continuous intracerebroventricular infusion for up to 20 days of two mAbs (RVC20 and RVC58) against the viral glycoprotein led to 56% survival of RABV-infected mice when initiated in the prodromal phase of the disease. Survival decreased to 33% when treatment was initiated one day later, in the early acute neurological phase. Direct administration of antibodies into the cerebrospinal fluid (CSF) is intended to bypass the BBB, but results in limited drug penetration into the brain parenchyma (48). The BBB remains a significant obstacle to the effective delivery of antivirals and immunotherapeutics for the treatment of rabies. Strategies to overcome this should be a part of drug discovery processes or added to the therapeutic regimen.

### Neuronal Degeneration

The molecular mechanisms that induce neuronal degeneration need to be managed in rabies patients. These mechanisms may be beneficial in early infection by impeding viral spread, but destructive once infection is more widespread. Deletion of the SARM1 gene significantly delayed axonal degeneration in RABV-infected neurons, from 24 h in the presence of the gene to beyond 7 days in SARM1 knockout neurons (33). In an *ex vivo* model of axonal damage using the mitochondrial respiratory chain inhibitor rotenone, which produces SARM1-dependent axonal degeneration, treatment with a SARM1 inhibitor not only prevented further axonal degeneration but also allowed recovery of axons that had already entered an intermediate stage of damage (49). These findings raise the possibility that modification of SARM1 function may be a novel ancillary therapeutic approach in rabies, in combination with other therapies. More broadly, enhanced understanding of rabies as a neurodegenerative disease may lead to application of neuroprotectants developed for other, more prevalent neurodegenerative diseases.

### Inflammation

Host immune responses are directed toward RABV and degenerate cellular material. These inflammatory processes may be lifesaving in early infection through the elimination of RABV, but damaging in later stages if widely activated in the CNS (50). A therapeutic benefit of immunomodulators in late RABV infection is supported by experimental studies in mice. When corticosteroid treatment was initiated early (0–24 h after inoculation) in mice inoculated with a bat RABV variant, mortality rates were 16% higher than in non-treated groups. However, if corticosteroid treatment was initiated later (72–96 h after inoculation), mortality rates were 14% lower in treated groups compared to controls, although this difference was not statistically significant, possibly due to the small sample size (51). More recently, Smreczak et al. (52) tested the effect of selected inhibitors of pro-inflammatory processes in RABV-infected mice, with treatment initiated from 5 days post-inoculation. Onset of clinical signs and time to death were delayed in mice treated with infliximab, an inhibitor of the pro-inflammatory cytokine TNF-α, or with sorafenib tosylate, a multi-kinase inhibitor. Treatment with tocilizumab, an IL-6 receptor blocker, also increased time to death, but this did not reach statistical significance. Conversely, Jackson et al. (53) showed that treatment with minocycline, a tetracycline derivative with anti-inflammatory properties, was not beneficial in adult mice infected with highly virulent RABV and was in fact detrimental in neonatal mice infected with an attenuated strain. In addition to differences due to RABV type (pathogenic vs. attenuated), host species may also influence immune responses to RABV infection. A recent study demonstrated that the inflammatory response in the mouse brain following RABV infection differed substantially from that in the human brain, with a more pronounced inflammatory response in the former (54), suggesting that the effect of immunomodulatory therapy in mice may not translate to humans. Rabid dogs naturally infected with wild-type RABV may have more similar CNS inflammatory responses to humans (55). Further studies should substantiate this as a basis to address the hypothesis of therapeutic benefit of immunomodulators in investigational protocols in rabid dogs.

### Systemic Compromise

An indispensable component of any therapeutic protocol for rabies will be aggressive critical care to manage the severe systemic compromise that occurs in patients in late-stage disease. Respiratory and cardiac complications are the most common (56). Critical care facilities will be needed to manage these complications, including mechanical ventilation and administration of supportive drugs (3, 4). Complications may result from the widespread CNS infection, involvement of the autonomic nervous system, or from extraneural organ involvement due to centrifugal spread of RABV from the CNS (57).

Timing of initiation of therapy for the four categories of disease process will be important, with expected improved outcomes from early initiation of therapy. Therapy should be tailored according to the stage of disease, clinical form (encephalitic vs. paralytic), immunological response, and patient prognosis. Tools for rapid, minimally-invasive bedside diagnostic and prognostic indicators are needed, and initiation of therapy prior to diagnostic confirmation will need to be considered in order to achieve optimal clinical outcomes. Neurofilament proteins from damaged neurons released into CSF and blood have emerged as sensitive biomarkers of neurodegeneration in several diseases (58, 59). Their utility in the diagnosis, prognosis and monitoring of response to therapy in rabies should be explored. Detection of microRNA signatures associated with RABV infection could also offer a novel diagnostic route and their value as markers of neuronal health and disease during rabies infection should be investigated (60).

## Innovation in Therapeutic Approaches

Preclinical studies of RABV infection are important for research and development of individual therapeutic components, particularly viral inhibitors and neuroprotectant drugs. However, preclinical animal models will not always replicate important aspects of the disease process in humans. Furthermore, rodent models do not easily allow for study of the effects of combination therapies that incorporate aggressive critical care, as this is not usually feasible in small animal models. Individual therapies that may only extend time to death in preclinical studies may have more substantial beneficial effects when combined and supported by aggressive critical care. Innovation and refinement of combination therapies for rabies could potentially be accelerated by systematic application in a patient population, but several factors reasonably preclude this investigational approach in human patients. The incidence of human cases is typically low in any given geographic area and may be unpredictable over time. Prediction of locations where outbreaks may occur is challenging, as these depend on external factors like incidence and vaccination rates in reservoir animal populations, and availability and uptake of human PEP. Higher numbers of human cases are generally restricted to resource-constrained areas, where dog vaccination may be limited and access to affordable PEP may not be readily available. In these circumstances, aggressive management of patients with investigational protocols, even if feasible, poses substantial ethical dilemmas for physicians, given the uncertainty of patient outcomes (7, 8).

We propose that development of a successful combination therapy for rabies could be accelerated by implementation of investigational protocols in naturally infected dogs, using a One Medicine approach. “One Medicine” is a term that captures the similarities between human and veterinary medicine and the benefit of collaboration in the study of pathogenesis and treatment of diseases that affect both humans and animals (https://www.cdc.gov/onehealth/basics/history/index.html). The etiology and pathogenesis of rabies are similar in dogs and humans, but the incidence is far higher and arguably more predictable in the former. Veterinary treatment of canine patients can, in many ways, mimic critical care and supportive therapy in human clinical scenarios. In endemic areas with robust veterinary surveillance and response systems, suspect rabid dogs are caught and killed by the veterinary services, thereby providing a potential point of access of canine patients to specialized critical care and investigational treatment on a compassionate use basis. In some of these areas, facilities and veterinary expertise in the critical care of dogs are well developed and available at a fraction of the cost for human intensive care patients. These factors create opportunities to initiate restraint, sedation, support, and critical care of canine rabies cases while investigating and refining therapeutic protocols, with dogs themselves standing to benefit through the compassionate use of investigational drugs. Even in the event of therapeutic failure, dogs would receive appropriate palliative care and euthanasia.

## Safety and Ethics of a One Medicine Approach to Rabies Treatment

Clinical care and investigational treatment of rabid canine patients requires careful consideration of risks and benefits to patients, clinical staff, and the public. Humane euthanasia and testing of brain specimens from suspect rabid animals reduce risk of RABV transmission and ideally provide timely information for the appropriate management of exposed persons and animals. Current antemortem diagnostic methods for rabies carry a relatively high probability of false negative results (61). Single negative antemortem test results cannot be relied on for decisions on whether to initiate or continue human PEP. Therefore, initiation of care and treatment of suspect rabid animals should be limited to those cases with no potential exposure of unvaccinated persons or should be coupled with timely provision of PEP to all potentially exposed persons regardless of case outcome.

Canine patients should be treated in specialized facilities that meet Animal Biosafety Level 2 (ABSL2) criteria (62). Protocols should include appropriate use of sedation and anesthesia to allow safe handling and enhance patient welfare. Routine precautions should be taken by clinical staff to prevent exposure to infectious material, particularly during procedures such as airway intubation, suctioning, and esophageal feeding tube placement. All staff should be fully vaccinated and undergo regular serological testing for RVNA levels. Clinical criteria such as euthanasia for therapeutic futility or discontinuation of ABSL2-level isolation should be established beforehand. Facilities and plans should be in place for post-therapeutic monitoring and rehabilitation of recovered patients, including monitoring for viral shedding over a predefined period.

## The Canine Rabies Treatment Initiative

**Figure 1:**
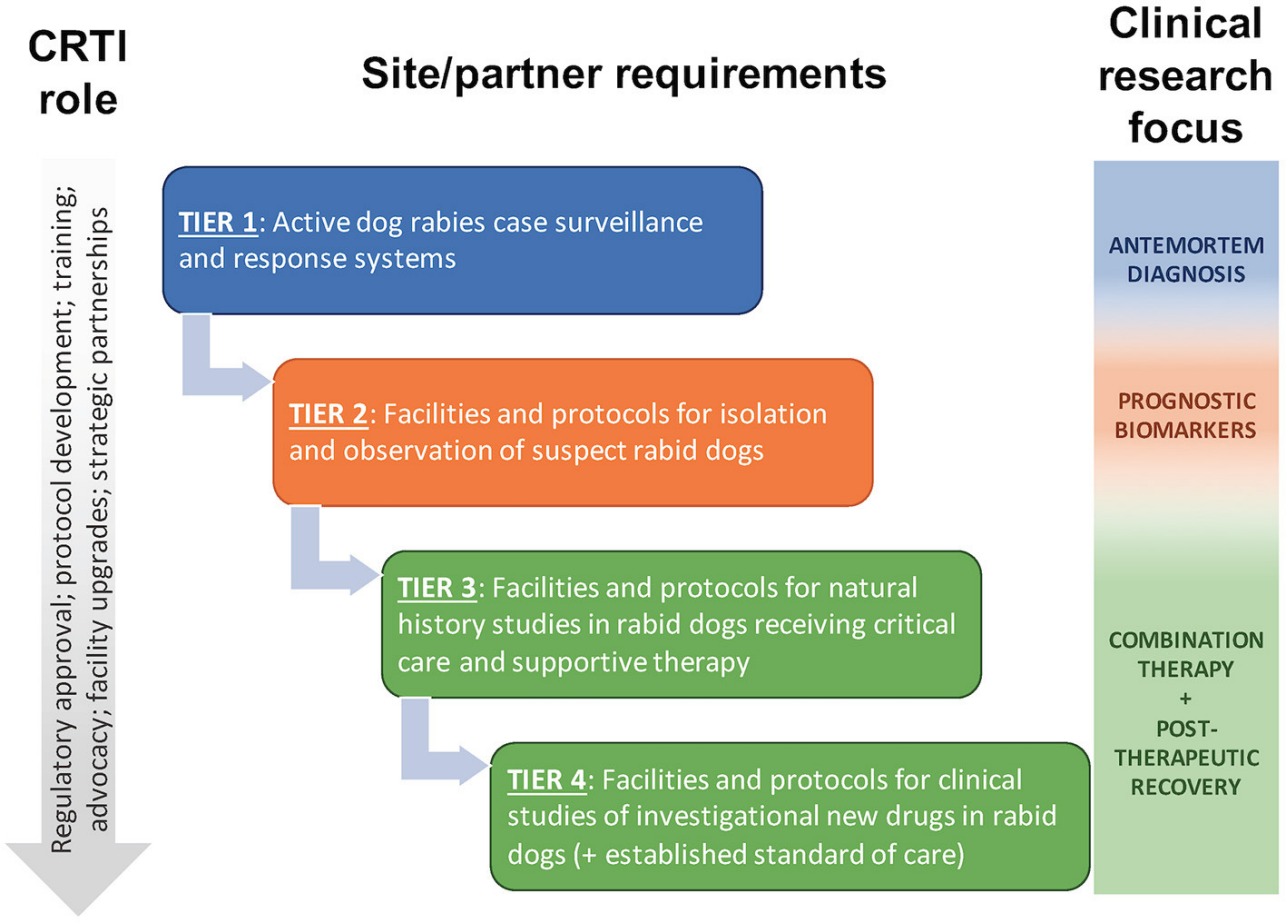
The Canine Rabies Treatment Initiative (CRTI) will implement a tiered approach to clinical research on rabies in naturally infected dogs, progressing to clinical studies of investigational new drugs in dogs receiving critical care and supportive therapy that mimic the human clinical scenario. Naturally infected dogs provide a higher and more predictable caseload than human patients, providing opportunities for additional clinical research to supplement preclinical and human clinical research.

The Canine Rabies Treatment Initiative (CRTI; www.treatrabies.org) is a non-profit organization established to attain the vision of rabies as a treatable disease for all patients. While we fully support efforts to eliminate human deaths from dog-mediated rabies through dog vaccination and human PEP, we believe there remains a need for an effective rabies therapy. The mission of the CRTI is to apply a One Medicine approach to clinical research on rabies in naturally infected dogs, as a model of the disease in humans and as patients in their own right, with the goal of developing an effective combination therapy for successful treatment of the disease. CRTI is implementing this mission through a tiered approach in canine rabies endemic areas (Figure 1), in collaboration with governmental agencies, non-governmental organizations and academic partners. This approach allows for phased progression toward treatment while conducting translational research on pathogenesis, diagnosis and prognosis; developing an ethical framework and clinical and biosafety protocols; providing training for veterinary personnel; obtaining regulatory approval; and strengthening strategic partnerships. Implementation of a One Medicine approach, with active involvement of veterinary clinician-scientists in the provision of compassionate care and investigational treatment to canine cases, will accelerate development of an effective combination therapy for rabies, ending the years of neglect of a treatment for this disease and providing hope for patients.

## Data Availability Statement

The original contributions presented in the study are included in the article/supplementary material, further inquiries can be directed to the corresponding author/s.

## Author Contributions

DK wrote the first draft of the manuscript. All authors contributed to manuscript revision, read, and approved the submitted version.

## Funding

HE receives funding for rabies research from a grant to Oxford University, UK, from the Wellcome Foundation. Open access publication fees were paid by the Center for Conservation Medicine and Ecosystem Health, Ross University School of Veterinary Medicine.

## Conflict of Interest

HE consults for several gene therapy companies (Freeline Inc, Takeda, Regenexbio, Biogen, Ring Therapeutics) and the Gamaleya Institute and holds equity in Virion Therapeutics. SM consults for two plasma collection/HRIG production companies (Kamada and BPL) and one animal rabies vaccine company (Elanco), advising on rabies serology techniques for vaccine immune response. CR was employed by LYSSA LLC. The remaining authors declare that the research was conducted in the absence of any commercial or financial relationships that could be construed as a potential conflict of interest.

## Publisher's Note

All claims expressed in this article are solely those of the authors and do not necessarily represent those of their affiliated organizations, or those of the publisher, the editors and the reviewers. Any product that may be evaluated in this article, or claim that may be made by its manufacturer, is not guaranteed or endorsed by the publisher.
